# Genome wide association study reveals novel QTL for barley yellow dwarf virus resistance in wheat

**DOI:** 10.1186/s12864-019-6249-1

**Published:** 2019-11-21

**Authors:** Shormin Choudhury, Philip Larkin, Rugen Xu, Matthew Hayden, Kerrie Forrest, Holger Meinke, Hongliang Hu, Meixue Zhou, Yun Fan

**Affiliations:** 1 Tasmanian Institute of Agriculture, University of Tasmania, Prospect, TAS Australia; 2 Department of Horticulture, Faculty of Agriculture, Sher-e-Bangla Agricultural University, Sher-e-Bangla Nagar, Dhaka, Bangladesh; 3 CSIRO Agriculture and Food, Canberra, Australia; 4 Barley Research Institution of Yangzhou University, Yangzhou University, Yangzhou, China; 5 School of Applied Systems Biology, La Trobe University, Bundoora, Victoria Australia; 6 Agriculture Victoria Research, AgriBio, 1 Park Drive, Bundoora, Victoria Australia

**Keywords:** Genome-wide association study, Novel QTL, SNP, BYD resistance, Wheat

## Abstract

**Background:**

Barley yellow dwarf (BYD) is an important virus disease that causes significant reductions in wheat yield. For effective control of *Barley yellow dwarf virus* through breeding, the identification of genetic sources of resistance is key to success. In this study, 335 geographically diverse wheat accessions genotyped using an Illumina iSelect 90 K single nucleotide polymorphisms (SNPs) bead chip array were used to identify new sources of resistance to BYD in different environments.

**Results:**

A genome-wide association study (GWAS) performed using all the generalised and mixed linkage models (GLM and MLM, respectively) identified a total of 36 significant marker-trait associations, four of which were consistently detected in the K model. These four novel quantitative trait loci (QTL) were identified on chromosomes 2A, 2B, 6A and 7A and associated with markers IWA3520, IWB24938, WB69770 and IWB57703, respectively. These four QTL showed an additive effect with the average visual symptom score of the lines containing resistance alleles of all four QTL being much lower than those with less favorable alleles. Several Chinese landraces, such as H-205 (Baimazha) and H-014 (Dahongmai) which have all four favorable alleles, showed consistently higher resistance in different field trials. None of them contained the previously described *Bdv2, Bdv3* or *Bdv4* genes for BYD resistance.

**Conclusions:**

This study identified multiple novel QTL for BYD resistance and some resistant wheat genotypes. These will be useful for breeders to generate combinations with and/or without *Bdv2* to achieve higher levels and more stable BYD resistance.

## Background

Barley yellow dwarf (BYD) is one of the most destructive wheat diseases worldwide and is caused by phloem limited luteoviruses recognised as barley yellow dwarf viruses (BYDV) [1]. The virus belongs to the *Luteoviridae* family and is transmitted by different aphid species. BYDV is divided into different distinct serotypes, based on the vector specificity and sequences of the virus. The most damaging serotype is BYDV-PAV [2], which is transmitted by the aphids *Rhopalosiphum padi* and *Sitobion avenae* [3].

Symptoms of BYDV infection in wheat vary among cultivars and environments with the major ones being leaf discoloration, reduced plant growth and grain yield. Yield losses in wheat are estimated to be 27–45 kg/ha for each 1% increase in BYDV incidence [4]. Yellow dwarf virus (YDV) disease can be partially controlled through management practices such as time of sowing and the application of insecticides. However, breeding for resistant or tolerant cultivars is the most efficient and environmentally sound approach to prevent yield losses [5].

Cereal crops are most vulnerable to BYDV infection during early growth stages. Yellowing or reddening of leaf blades along the vascular bundles, especially at the leaf tips, and plant dwarfing, are the main symptoms of YDV disease in wheat [6, 7]. These symptoms are positively correlated with the virus titre as measured by enzyme-linked immunosorbent assay (ELISA), which is an indicator for disease susceptibility [8]. In resistant plants, virus multiplication is reduced. The evaluation of wheat for BYD resistance using aphid inoculation and ELISA analyses is both laborious and costly. Marker assisted selection (MAS) of known resistance loci would allow quicker progress in introgressing resistance loci into elite lines and breeders’ germplasm.

Genetic mapping of bi-parental populations using molecular markers has been used to identify and characterise a number of QTL in common wheat for BYDV-PAV resistance. These include 22 QTL identified from the Opata × Synthetic recombinant inbred population (RIL) population and seven QTL from the Frontana × INIA66 RIL population [9] with one of the QTL from the Frontana × INIA66 population being located on 7DS at the same position of the *Bdv1* gene identified from a wheat cultivar Anza [10]. In addition, three BYD resistance genes from *Thinopyrum intermedium* (intermediate wheatgrass), called *Bdv2*, *Bdv3* and *Bdv4*, have been introgressed into common wheat background via chromosomal translocations [11]. *Bdv2* was first introduced as a 7D-7Ai#1 translocation [4], *Bdv3* as a 7B-7Ai#1 translocation [12] (Crasta et al. 2000) and *Bdv4* as a 2D-2Ai-2 translocation [11]. Evidently the different BYD resistance genes in *Th. intermedium* have different isolate specificities [13] and possibly diverse mechanisms of action such as interfering with virus multiplication [14] or reducing cell-to-cell movement [15].

While effective sources of resistance to BYD have been identified in wheatgrasses, few have been reported in common wheat [16]. The only reported gene for BYD resistance in wheat, *Bdv1*, and associated with the rust resistance *Lr34/Yr18* gene complex, may reduce leaf symptoms [10] but fails to confer any protection for biomass or grain yield [9, 16]. In this study we performed a genome-wide association study on a geographically diverse collection of 335 bread wheat accessions to identify new sources of resistance to BYD.

## Results

### BYD resistance of wheat accessions

Visual symptom scoring (VSS) was performed at the heading stage when most of the sensitive varieties (e.g., Revenue, Yu-1) revealed prominent visual symptom. Resistance scores showed a high level of variation among trials and replicates, especially among the susceptible accessions (Additional file 1: Figure S1). This led to low correlations (even though significant) (Additional file 2: Figure S2) between trials and low heritability of VSS (h_B_^2^ = 0.11). For examples, Yu-10, a sensitive genotype, ranged from 0 (no symptom) to 5 (very susceptible) across different trials and replicates. However, some resistant lines showed resistance in all the trials/replicates, with no symptom scores being over 2. This variability is not surprising since BYDV infection relies on both aphid spread and the proportion of highly viruliferous aphids in the population, which are extremely variable under field conditions. Thus, it is crucial to obtain phenotypic data from multiple trials. In this study, the average scores of three trials over 2 years were used for further analysis. Of all the accessions, 2% were resistant (VSS = 0–1), 33% moderately resistant (VSS ≥1–2), 61% susceptible (VSS ≥2–3) and 3% highly susceptible (VSS ≥3) to BYDV.

To determine if any of the resistant genotypes contained *Bdv2* or *Bdv3* genes carried on the group 7 translocations from *Th. intermedium*, several markers linked to the translocations [17, 18] were used to screen the entire population. Known *Bdv2* containing wheat varieties Mackellar, Manning and Zhong4 [19, 20] were used as positive controls. Except for these positive controls and XuBYDV, a breeding line from China, all other genotypes in the collection showed a different amplicon size, indicating the absence of *Bdv2* or *Bdv3* genes (Additional file 3: Figure S3).

### Association mapping for BYD resistance

**Fig. 1:**
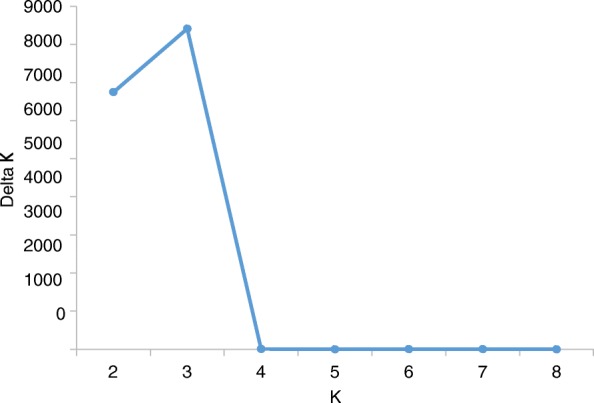
An estimation of the most probable number of clusters (K), based on 20 independent runs and K ranging from 2 to 8

**Fig. 2:**
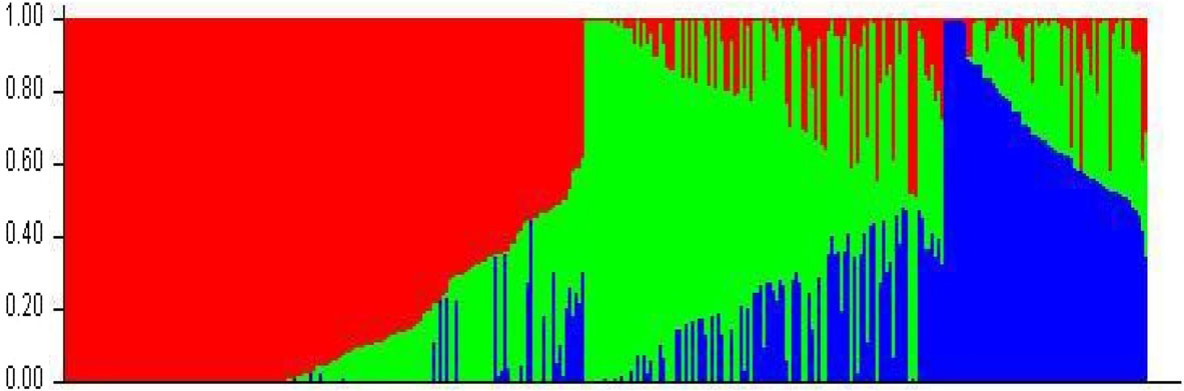
Population structure of the 335 wheat accessions. Three subpopulations (K = 3) were produced on genetic diversity detected by 4560 SNP markers, each are presented by a different colour

The 335 wheat accessions were used to analyse the population structure. To determine the most probable number of subpopulations among all accessions, the largest value of statistic index ΔK was used as an indicator [21]. In this study, ΔK reached its highest value when K = 3 (Fig. 1), suggesting the accessions were comprised of three subpopulations. Details of the subpopulation structure for each of the 335 wheat accessions are shown in Fig. 2 and listed in Additional file 4: Table S1.

**Fig. 3:**
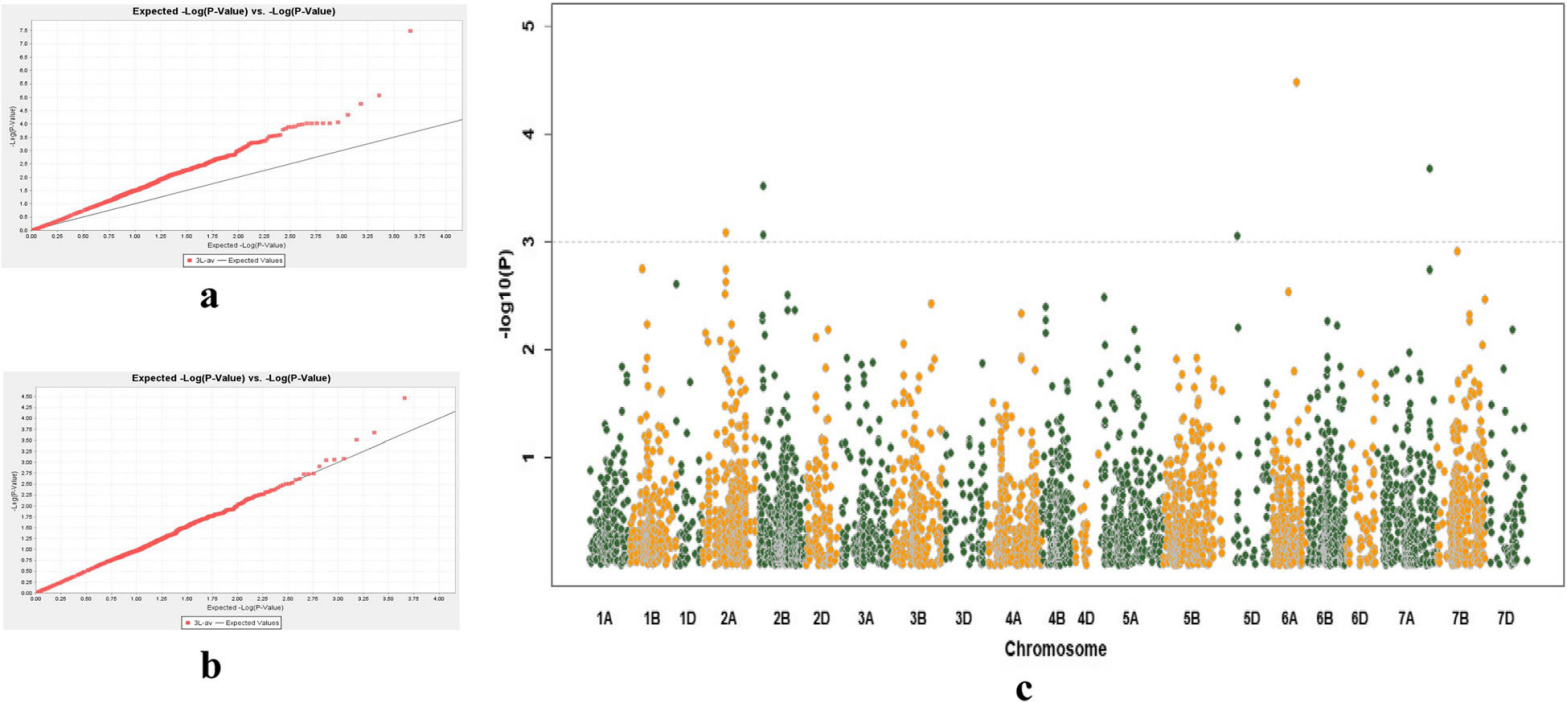
Manhattan plots and Quantile-quantile (Q-Q) plots for genome wide association study (GWAS) of BYD resistance in 335 wheat accessions. **a** Manhattan plot in Q method; **b** Manhattan plot in K method; **c** Q-Q plot in K method. In Manhattan plots, significant association was identified using criterion of –log10 (P) > 3 (*P* < 0.001). Q-Q plots were displayed in marker–trait association analysis. The black line represents the expected line under the null distribution, while the red symbol in the observed –log10 (P) for BYD resistance

The fitness and efficiency assessment of different models by Quantile-Quantile (Q-Q) plot indicated that the observed –log_10_ (P) values for BYD resistance were closer to expected –log_10_ (P) values in the K (Fig. 3b) method than those from the Q (Fig. 3a) method. Thus only the K method was used to identify the QTL. Criteria for significant marker-trait association was set for *P*-value < 0.001. Four significant marker trait associations were detected with the K method, with the QTL being located on 2A (IWA3520, 276.89 cM), 2B (IWB24938, 82.22 cM), 6A (IWB69770, 284.1 cM) and 7A (IWB57703, 624.47 cM) (Fig. 3c; Table 1).

**Table 1:** Association mapping results for BYD tolerance using the K method

Trait	Chromosome	Position (cM)	Marker	*P*	Marker *R*^2^
BYDV	2A	276.89	IWA3520	8.28E-04	0.044
BYDV	2B	87.22	IWB24938	3.10E-04	0.052
BYDV	6A	284.1	IWB69770	3.40E-05	0.074
BYDV	7A	624.47	IWB57703	2.13E-04	0.056

BYDV, *barley yellow dwarf virus* resistance data are averaged of three trials over two growth seasons

Due to the low correlations and low heritability of VSS, further analysis was conducted using the best linear unbiased prediction (BLUP) method. There was consistency between GWAS results from the average data of three trials and those from BLUP (Additional file 5: Table S2). However, the LOD value of all the significant SNPs were generally lower when GWAS was based on BLUP.

**Fig. 4:**
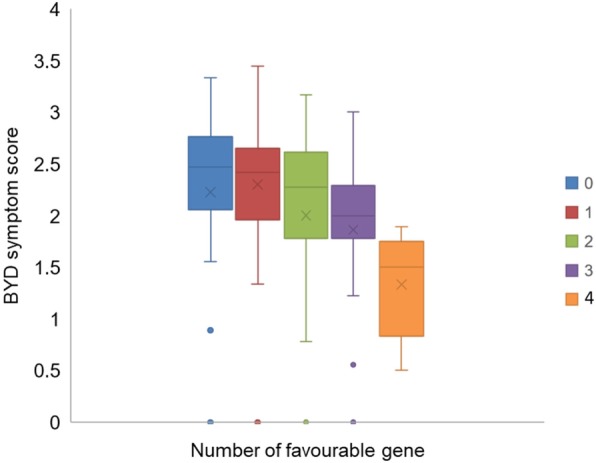
Average symptom scores of genotypes with resistance alleles. 0: without any tolerance alleles, 1–4: with 1–4 resistance alleles

The average symptom scores for genotypes carrying different combinations of the resistance alleles is shown in Fig. 4. The effect of the resistance alleles appeared to be additive since the average score decreased in genotypes that carried an increasing number of resistance alleles.

## Discussion

Barley yellow dwarf is one of the most important viral diseases of cereals worldwide and can lead to substantial yield losses that could potentially threaten food security [22]. Available resistances to BYD in wheat are only partially effective but can be pyramided using marker assisted breeding to achieve higher levels of resistance [13, 23]. To further enhance resistance to BYDV in common wheat, the identification of new sources of BYD resistance is important. In this study, 335 common wheat accessions were assessed for BYD resistance and a genome wide association study to identify genomic regions for BYD resistance was performed. Four markers were identified to have consistent and significant associations with BYD resistance.

The leaf symptoms found in BYDV-infected plants are commonly used to select BYD resistance in cereal crops [7, 24, 25]. Most of the genotypes tested in this study showed symptoms of BYDV infection, which was confirmed by detection of the virus using ELISA (data not shown). The scores of the symptoms were used to identify associated QTL. Of the three models used in this study, the Q model showed more associations than the K model. This was expected from previous work showing that mixed linear model (MLM) typically detects fewer QTL than general linear model (GLM) models because the use of both the Q and K matrixes helps to reduce confounding factors such as population structure [26, 27]. Using the K method, four QTL were identified. These QTL were located on 2A (276.89 cM), 2B (87.22), 6A (284.1 cM) and 7A (624.47 cM), respectively (Fig. 3; Table 1). By comparing the position of associated markers with the consensus SNP map, the QTL for BYD resistance on chromosome 2B is at a different position to that identified for BYDV induced dwarfism and biomass reduction in the wheat population Opata × Synthetic [9]. The QTL on 6A (284.1 cM) with the nearest marker IWB69770 was located at a different position (long arm) to the reported QTL for yellowing [9]. No QTL for BYD resistance was reported by Ayala et al. [9] on chromosome 2A and 7A, which were identified in the current study. These four QTL showed an additive effect with the average visual symptom score of the lines containing resistance alleles of all four QTL being much lower than those with less favourite alleles (Fig. 4).

Few wheat cultivars are reported to have a high level of BYD resistance [25]. The current three known BYD resistance genes used for breeding or research, *Bdv2*, *Bdv3* and *Bdv4*, are all on translocations from *Th. intermedium* (intermediate wheatgrass) [11]. Zhong 4 is a partial amphiploid between wheat and *Th. intermedium*, 2n = 56, with 7 pairs of chromosomes added from *Th. intermedium* [28]. Zhong 4 is known to have BYD resistance genes on both a group 2 and a group 7 wheatgrass chromosome [29, 30] whose combined effect is strong resistance [23]. Zhong 4 was added to the current study as a resistant control and showed a consistently low level of infection in all trials. BYD resistance was also identified from wheat germplasm with some showing a similar to or even better BYD resistance than Zhong 4. Most of these resistant genotypes are from China which included XuBYDV (a breeding line from Beijing, China), H-014 (Dahongmai, a landrace from Shanxi, China), H-151 (Sanyuehuang, a landrace from Jiangsu, China), H-20 (Baiqimai, a landrace from Gansu, China), H-205 (Baimaza, a landrace from Ningxia, China), H-027 (Hongpidongmai, a landrace from Shanxi, China), H-023 (Daimanghongmai, a landrace from Tianjin, China) and H-056 (Shuilizhan, a landrace from Jiangxi, China). After screening the population with a *Bdv2* or *Bdv3* specific marker, *SSR-Bdv3* [17], it was confirmed that none of these resistant genotypes contained the *Bdv2 or Bdv3*. Furthermore *Bdv4* is known only as a 2D-2Ai-2 centric fusion, and none of the QTL of this study were on 2D, allowing the conclusion that it was not present in the population. This opens the opportunity of discovering new BYD resitance genes associated with the new QTL. Further bi-parental populations will be produced to confirm and undertake fine mapping of the new QTL for BYD resistance in wheat.

A total of 224 annotated genes were identified in around 10 Mbp of genomic sequence corresponding to the QTL intervals in chromosomes 2A, 2B, 6A and 7A (Additional file 6: Table S3). Among these, eight candidate genes were predicted to relate to plant defense in different species based on published results. Three candidate genes were found for the 2 Mbp QTL interval on chromosome 2B which encode: a receptor kinase-like protein (TraesCS2B01G037300) which mediates disease resistance by activating cellular defense response [31]; a subtilisin-like protease (TraesCS2B01G038300), a protein family associated with plant defense responses to biotic stress including modification of cell wall and programmed cell death [32]; and a glycine-rich protein (TraesCS2B01G038200), a protein family associated with plant defense mechanism [33]. For the major QTL on chromosome 6A, there were five candidate genes in the 2.4 Mb QTL interval. Among these TraesCS6A01G368200 and TraesCS6A01G368400 encode peroxidases which play a pivotal role in chemical defense mechanisms that control the development of virus disease in many plants [34–36]. TraesCS6A01G367000.1 and TraesCS6A01G367000.2 encode bifunctional nucleases involved in basal defense response, participating in abscisic acid-derived callose deposition following infection by a necrotrophic pathogen [37]. TraesCS6A01G367800 encodes a leucine-rich repeat protein kinase which acts as as a contributor to basal defense against *Fusarium* head blight and as an upstream component of salicylic acid signaling in wheat [38]. These candidate genes can be selected as target genes in future study.

## Conclusion

This is the first GWAS study that utilize the wheat iSelect 90 K SNP array to explore BYD resistance QTL. A total of four significant QTL were identified. Some of the genotypes in the study showed similar or even better resistance to BYD than those genotypes with known resistance *Bdv2* but contained resistance genes different from *Bdv2*. Most of the resistant lines are Chinese landrace. With further characterisation, these lines and the four identified QTL will be useful for breeders to generate combinations with and/or without *Bdv2* to achieve higher levels and more stable BYD resistance.

## Methods

### Aphid Colony

Bird-cherry aphid, *Rhopalosiphum padi*, was collected from a Tasmanian barley field trial in 2014 and raised on oat (cv. Eurabbie-a BYDV susceptible genotype) in small cages at 20 °C ± 2 °C, 65 ± 5% RH, with a photoperiod of L14:D10 by cool white fluorescent light under 450 μmol.m^− 2^ s^− 1^ photosynthetically active radiation.

### Collection and maintenance of virus isolate

The isolate of BYDV-PAV was obtained from the University of New England, New South Wales (NSW), Australia and maintained in oat cv Eurabbie in small cages under the similar conditions as per the aphid colonies. The virus isolates were periodically (8-weekly) moved to new plants with *R. padi*. To ensure viruliferous aphid colonies, the infection status was frequently tested using ELISA which uses polyclonal BYDV-PAV antibodies [39]. Results were assessed by using a Multiskan RC plate reader with GENESIS software (Lab Systems). All samples were tested twice. Samples with absorbance values greater than twice the mean of negative controls in one or both samples were considered positive.

### Plant materials, virus inoculation and phenotyping

**Fig. 5:**
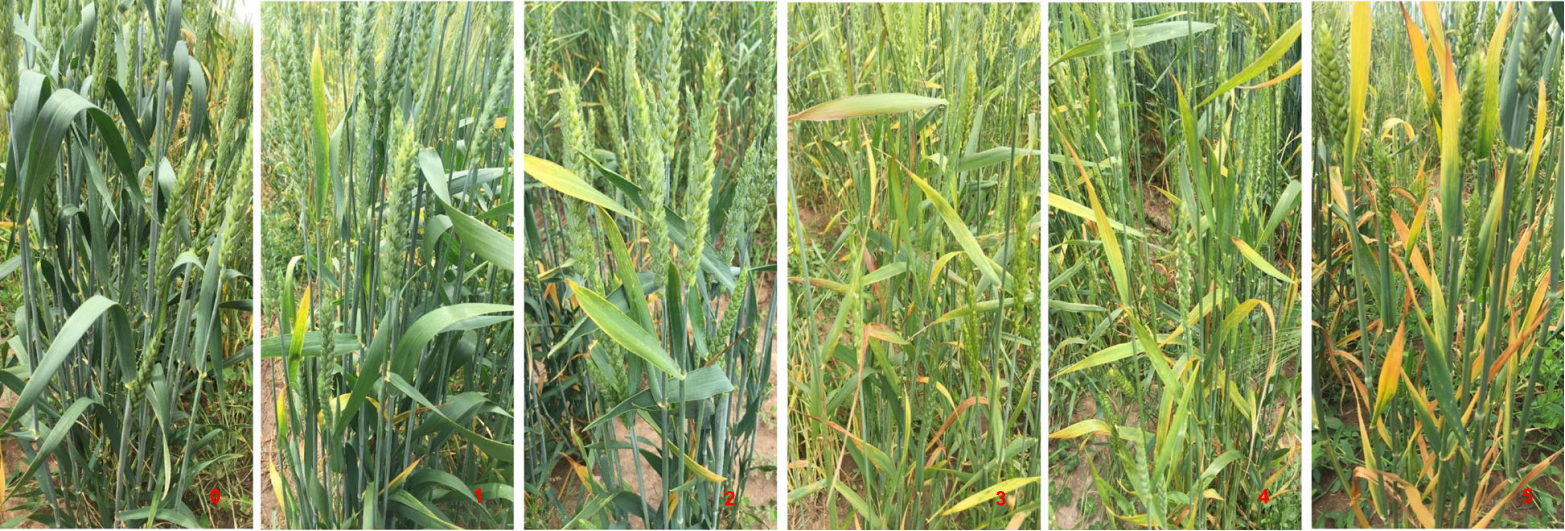
BYD symptom severity scores in wheat. Score 0, whole plant without symptoms; score 1, few leaves showing discoloration; score 2, about 20% leaf area has discoloration; score 3, 40% leaf area shows yellowing; score 4, 60%, leaf area shows yellowing; score 5, most of plant affected (photos were taken from field trials of the current experimental)

A total 335 wheat accessions obtained from China and the Australian Grains Genebank were used in this study. These accessions were evaluated for BYD resistance in 2016 and 2017 at the Tasmanian Institute of Agriculture, Launceston, Australia. Each accession was grown in triplicate in hill plots in a randomised complete block design. Five seeds were sown in each hill plot. Each hill plot was inoculated at the 2-leaf stage (Zadok’s Growth Stage 12) with BYDV-PAV using ten to fifteen viruliferous adult aphids (*R. padi*) [6]. An inoculation access period of 120 h was used to ensure virus infection of all plants before aphids were killed by spraying 1 ml/L solution of the insecticide Karate (Syngenta Ltd.). The accessions were also evaluated in the field in 2017 under natural BYDV infection at the Burlington Road at Cressy Research Station, Tasmania (− 41.709400 N, 147.094400 E). Each accession was sown in triplicate in 50 cm rows, with 15 seeds per row and a row spacing of 40 cm, using a randomised block design. Standard agronomic management practises were used to maintain each of the three trials. Disease severity was scored for BYD visual symptoms at heading stage on a 0–5 scale, where 0 = no visible BYD symptoms, 1 = few discoloured leaves scored, 2 = plants had approximately 20% of leaves affected, 3 = 40% of leaves affected, 4 = 60% of leaves affected, and 5 = almost all the plant affected (Fig. 5).

### Genotyping

DNA was extracted from leaf tissue collected at the 2-leaf seedling stage from a single plant per accession and genotyped using the Illumina iSelect 90,000 SNP bead chip assay described in [40]. Genome Studio polyploid clustering V1.0 software (Illumina Ltd.) was used to export normalized NormR and Theta values for each accession for SNPs that produced well-separated clusters for unambiguous scoring and had been previously genetically mapped [40]. SNP genotype calling was performed using a custom PERL script that assigned a genotype to each accession based on the Euclidian distance of the sample data point to the centre of pre-defined clusters having known allelic relationships, considering the standard deviations of the defined clusters. A total of 38,379 SNPs were identified to be polymorphic in the population. The SNP markers with a less than 90% call rate across samples, a minor allele frequency less than 0.05, or that were redundant were removed. A final number of 4560 SNPs were used for population structure and kingship analysis. LOD values for significant levels of *p* < 0.05 ranged from 2.4–3.2 for different chromosomes.

### Population structure and kinship analysis

The population structure of the association mapping panel was assessed using all 4560 SNP markers, which were distributed across the 21 wheat chromosomes, using the software STRUCTURE v2.3.3 [41].. The number of underlying subpopulations was determined from the largest value of the ΔK statistic [21]. The number of clusters (K) varied from 2 to 8 and 10 iterations were conducted in an admixture model with a 10,000 burning period and 10,000 MCMC (Markov Chain Monte Carlo). K value was the number of clusters when ∆K achieved maximum value [21]. SPAGeDi software was used to conduct a kinship analysis [42]. The kinship matrix measured the genetic similarity between individuals.

### Genome wide association study

The software TASSEL v3.0 was used to conduct association mapping of BYD resistance in wheat. Information on SNP markers (genotype), population structure, kinship and phenotype traits were imported into TASSEL 3.0. The following models were used for GWAS: (i) Q model, a general linear model (GLM) which sets the Q matrix as a fixed effect; and (ii) K model, a model which sets the kinship matrix as a random effect among genotypes. In the association study, the thresholds were determined as a significant level of *P* < 0.001 (−log_10_ (P) > 3). Manhattan plots were drawn using R software (v2.14.2). Quantile-quantile (Q-Q) plots implemented in TASSEL v3.0 were used to evaluate the fitness and efficiency of these models. After fitness and efficiency assessment, K model was selected to identify significant association between markers and BYD resistance.

### Detection of BYD resistant genes through Polymerase Chain Reaction (PCR)

Four markers which are reported to be closely linked to *Bdv2* or *Bdv3* genes were used to assess the presence or absence of the tolerance genes in wheat germplasm. The details of the primer pairs for these markers are listed in Table 2. A PCR was performed in 20 μL reaction mixtures containing approximately 15 ng of gDNA, and 0.4 μM of each species-specific forward and reverse primers. The amplification reactions were performed using the PCR system (Bio-Rad T100TM Thermal cycler, USA). The amplification conditions were as follows: initial denaturation at 95 °C for 2 min, followed by 35 cycles of 95 °C for 30 s, 57 °C for 30 s, and 72 °C for 30 s, and a final extension step at 72 °C for 5 min. To verify the PCR results, PCR products were resolved by 1.5% agarose gel electrophoresis. After electrophoresis, the gels were documented under UV using gel documentation system (Bio-Rad, USA).

**Table 2:** List of markers with chromosomal location used for identifying BYD resistance gene in wheat

Marker type	Primer	R gene	Primer sequence		
			Forward (5′ - 3′)	Reverse (5′ - 3′)	Reference
SCAR	BYAgi	*Bdv2*	ACT TCA TTG TTG ATC TTG CAT G	CAT GGA TAA TTC AGG GAG CAT TCT G	[43]
SCAR	AD2	*Bdv2*	TGA ACC GCT TCC AGT AAT GGA C	CTG AAC CGC TTC AGC GGT TCA G	[23]
SSR	Xgwm37	*Bdv2*	ACT TCA TTG TTG ATC TTG CAT G	CGA CGA ATT CCC AGC TAA AC	[44]
SSR	Bdv3	*Bdv2*/ *Bdv3*	CGA CGA ATT CCC AGC TAA ACT AGA CT	CTT AAC TTC ATT GTT GAT CTT A	[17]

### Functional annotation of putative genes in the region of QTL for BYD resistance

To analyse the biological functions of putative genes associated with BYD resistance, we performed a functional annotation of around 2 Mb physical nucleotide interval of significant SNP markers of each QTL. The sequences of significant SNP markers were blasted on https://wheat-urgi.versailles.inra.fr/ and https://urgi.versailles.inra.fr/blast/?dbgroup=wheat_whole_genome_assemblies&program=blastn. Annotated functions in wheat were downloaded from https://urgi.versailles.inra.fr/download/iwgsc/IWGSC_RefSeq_Annotations/v1.0/

## Supplementary information

**Additional file 1: Figure S1.** Distribution of BYD symptom scores of selected genotypes over different trials/replications.

**Additional file 2: Figure S2.** Correlations between different trials for visual symptom scoring.

**Additional file 3: Figure S3.** PCR products of BYD resistance gene (*Bdv2*) congaing cultivars (Mackellar and Manning) and resistant lines (H-014, H-020, H-039, H-096) amplified with the SSR primer of Bdv3.

**Additional file 4: Table S1.** The value of population structure of 335 genotypes.

**Additional file 5: Table S2.** Comparison of association mapping results for BYD tolerance using the K model, based on the average data from three trials and the data using the best linear unbiased prediction (BLUP) method.

**Additional file 6: Table S3.** Results of the putative candidate genes associated with BYD resistance on chromosome 2A, 2B, 6A and 7A.

## Data Availability

All supporting data can be found within the manuscript and its additional files.

## Abbreviations

BYD: Barley yellow dwarf
BYDV: Barley yellow dwarf virus
ELISA: Enzyme-linked immunosorbent assay
GLM: Generalised linear model
GWAS: Genome-wide association study
K: Number of clusters
LD: Linkage disequilibrium
LOD: Logarithm of odds
MLM: Mixed linear model
PCR: Polymerase Chain Reaction
Q: Population structure
QQ: Quantilequantile
QTLs: Quantitative trait locus
R2: Percentage of phenotypic variation explained by the identified SNPs
SNP: Single nucleotide polymorphisms
VSS: Visual symptom score
