# Supplementary material for: Genome wide association study reveals novel QTL for barley yellow dwarf virus resistance in wheat
Source: BMC Genomics. 2019 Nov 21;20:891. doi: 10.1186/s12864-019-6249-1 (PMC6873737; doi:10.1186/s12864-019-6249-1)
Supplement: Supplementary file 1 — Additional file 1: Figure S1. Distribution of BYD symptom scores of selected genotypes over different trials/replications. [file 12864_2019_6249_MOESM1_ESM.pptx]

## Slide 1
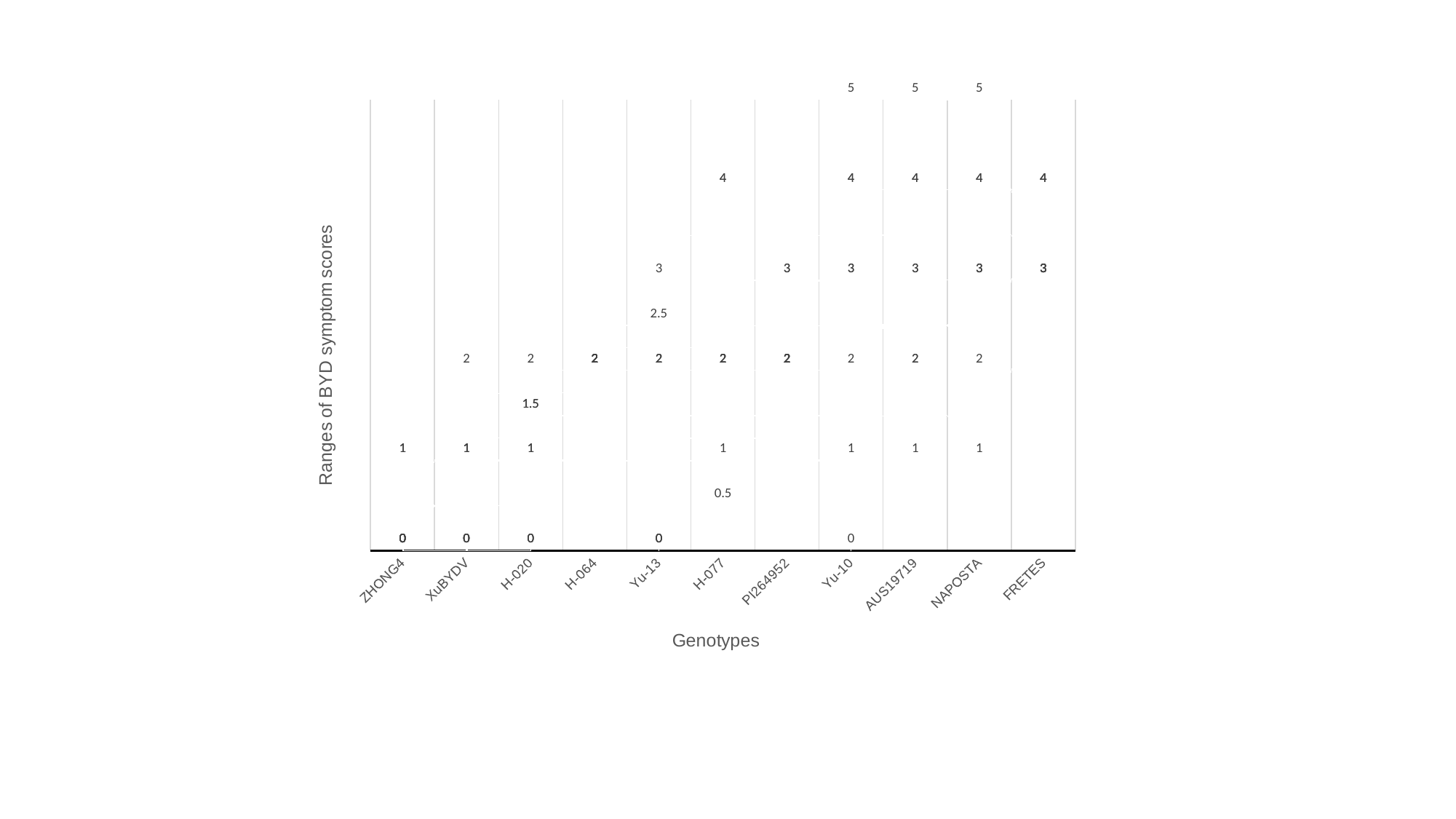

### Chart
| Category | | | | | | | | | |
|---|---|---|---|---|---|---|---|---|---|
| ZHONG4 | 1.0 | 1.0 | None | 0.0 | 0.0 | 0.0 | 0.0 | 0.0 | 0.0 |
| XuBYDV | 0.0 | 0.0 | None | 0.0 | 1.0 | 2.0 | 1.0 | 1.0 | 0.0 |
| H-020 | 0.0 | 0.0 | None | 2.0 | 1.5 | 1.5 | 0.0 | 1.0 | 1.0 |
| H-064 | 2.0 | 2.0 | None | 2.0 | 2.0 | 2.0 | 2.0 | 2.0 | 2.0 |
| Yu-13 | 2.0 | 2.0 | None | 2.0 | 0.0 | 3.0 | 0.0 | 2.5 | 0.0 |
| H-077 | 2.0 | 1.0 | None | 0.5 | 2.0 | 4.0 | 4.0 | 2.0 | 2.0 |
| PI264952 | 2.0 | 2.0 | None | 2.0 | 3.0 | 2.0 | 3.0 | 2.0 | 2.0 |
| Yu-10 | 1.0 | 3.0 | None | 0.0 | 4.0 | 4.0 | 5.0 | 3.0 | 2.0 |
| AUS19719 | 2.0 | 2.0 | None | 5.0 | 4.0 | 3.0 | 1.0 | 4.0 | 3.0 |
| NAPOSTA | 3.0 | 1.0 | None | 5.0 | 4.0 | 3.0 | 3.0 | 4.0 | 2.0 |
| FRETES | 3.0 | 3.0 | None | 3.0 | 3.0 | 3.0 | 4.0 | 4.0 | 4.0 |
