# Supplementary figures and images for: Genome wide association study reveals novel QTL for barley yellow dwarf virus resistance in wheat
Source: BMC Genomics. 2019 Nov 21;20:891. doi: 10.1186/s12864-019-6249-1 (PMC6873737; doi:10.1186/s12864-019-6249-1)

## Slide 1
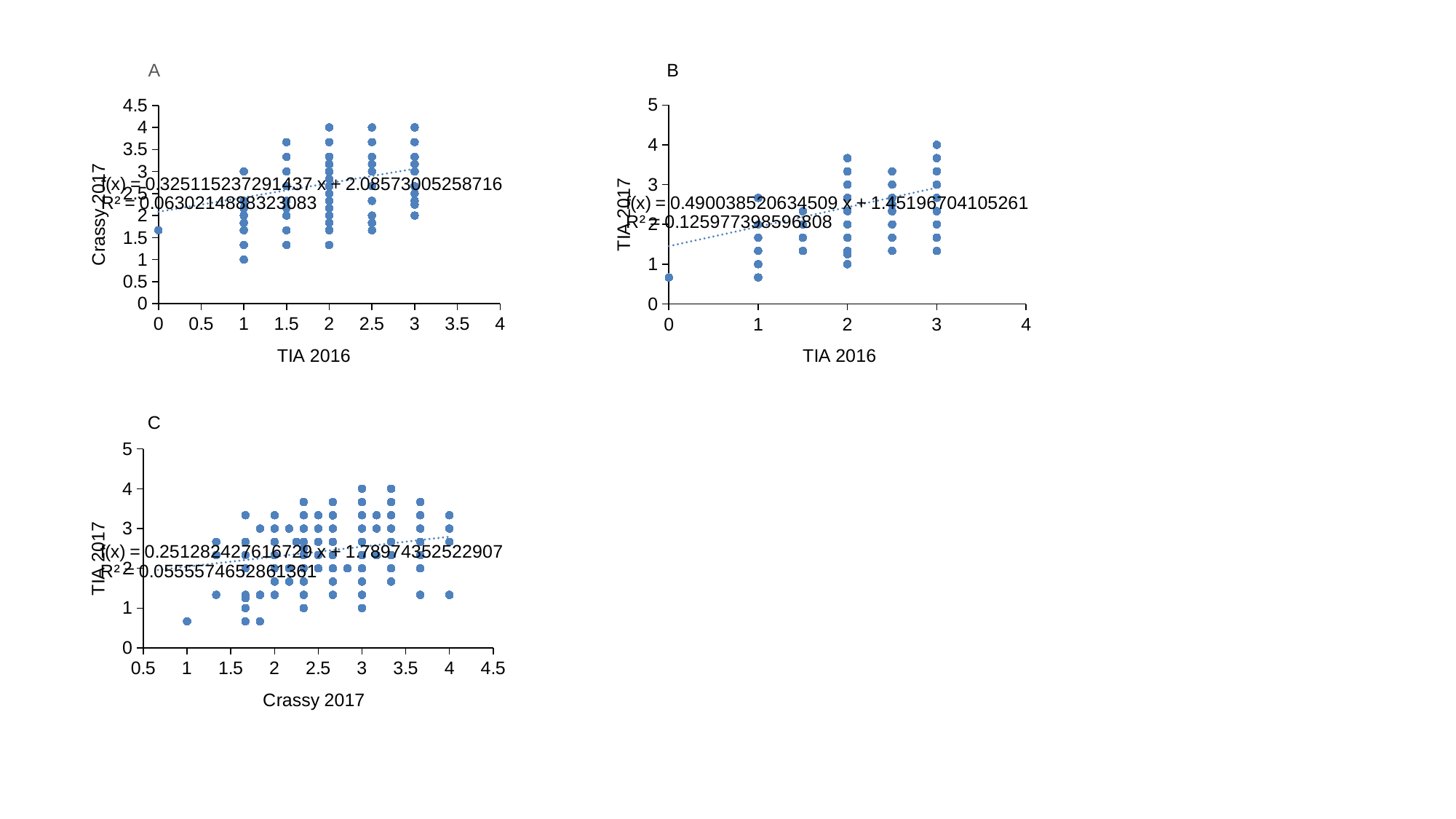

### Chart: A
| Category | |
|---|---|
### Chart: B
| Category | |
|---|---|
### Chart: C
| Category | |
|---|---|

Supplement: Supplementary file 2 — Additional file 2: Figure S2. Correlations between different trials for visual symptom scoring. [file 12864_2019_6249_MOESM2_ESM.pptx]

## Slide 1
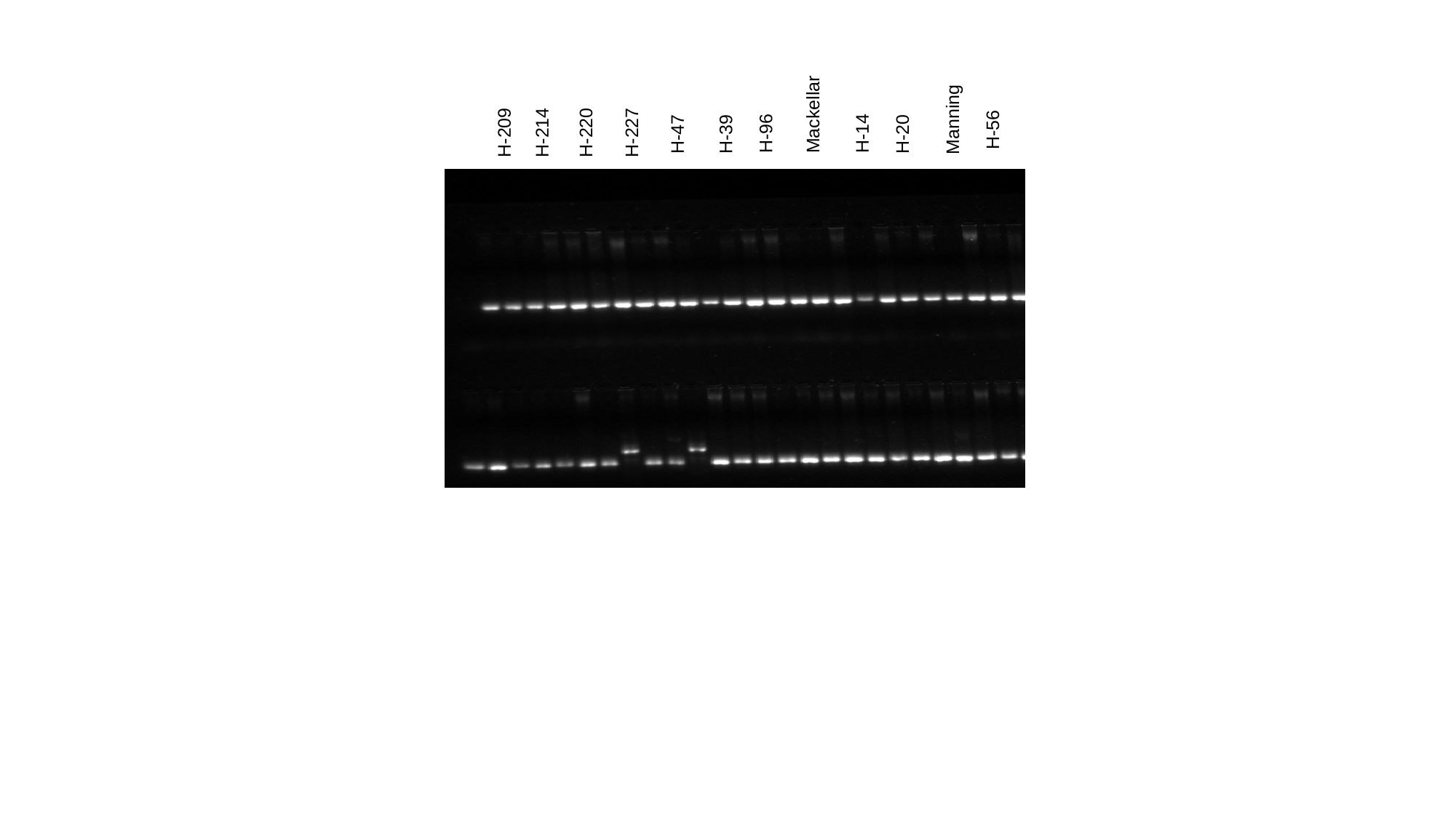

Mackellar
Manning
H-56
H-209
H-214
H-220
H-227
H-96
H-39
H-47
H-20
H-14

Supplement: Supplementary file 3 — Additional file 3: Figure S3. PCR products of BYD resistance gene (Bdv2) congaing cultivars (Mackellar and Manning) and resistant lines (H-014, H-020, H-039, H-096) amplified with the SSR primer of Bdv3. [file 12864_2019_6249_MOESM3_ESM.pptx]
